# Exposure Time Dependence of Operators’ Head Entrance Air Kerma in Interventional Radiology Measured by TLD-100H Chips

**DOI:** 10.3390/s25123666

**Published:** 2025-06-11

**Authors:** Rocco Mottareale, Francesco Manna, Patrizio Antonio Carmosino, Francesco Fiore, Marco Correra, Salvatore Stilo, Luca Tarotto, Mariagabriella Pugliese

**Affiliations:** 1 Department of Physics “E. Pancini”, Federico II University, 80126 Naples, Italy; rocco.mottareale@unina.it (R.M.); mpuglies@na.infn.it (M.P.); 2 Centro Servizi Metrologici e Tecnologici Avanzati, Federico II University, 80146 Naples, Italy; 3 Interventional Radiology Unit, Istituto Nazionale Tumori IRCCS Fondazione G. Pascale, 80131 Naples, Italy; pa.carmosino@istitutotumori.na.it (P.A.C.); f.fiore@istitutotumori.na.it (F.F.); m.correra@istitutotumori.na.it (M.C.); salvatore.stilo@istitutotumori.na.it (S.S.); luca.tarotto@istitutotumori.na.it (L.T.); 4 National Institute of Nuclear Physics, Section of Naples, 80126 Naples, Italy

**Keywords:** radiation protection, ionizing radiations, interventional radiology, thermoluminescent dosimetry

## Abstract

Interventional radiology offers minimally invasive procedures guided by real-time imaging, reducing surgical risks and enhancing patient recovery. While beneficial to patients, these advancements increase occupational hazards for physicians due to chronic exposure to ionizing radiation. This exposure raises health risks like radiation-induced cataracts, cardiovascular disease, and cancer. Despite regulations like the European Council Directive 2013/59/EURATOM, which sets limits on whole-body and eye lens doses, no dose limits exist for the brain and meninges, since the brain has traditionally been considered a radioresistant organ. Recent studies, however, have highlighted radiation-induced brain damage, suggesting that meningeal exposure in interventional radiology may be underestimated. This study evaluates the entrance air Cumulative mean annual entrance air kerma to the skullull during interventional radiology procedures, using thermoluminescent dosimeters and controlled exposure simulations. Data were collected by varying the exposure time and analyzing the contribution to the entrance air kerma on each side of the head. The results indicate that, considering the attenuation of the cranial bone, the absorbed dose to the brain, obtained by averaging the head entrance air kerma for the right, front, and left sides of the operator’s head, could represent 0.81% to 2.18% of the annual regulatory limit in Italy of 20 mSv for the average annual effective dose of exposed workers (LD 101/2020). These results provide an assessment of brain exposure, highlighting the relatively low but non-negligible contribution of brain irradiation to the overall occupational dose constraint. Additionally, a correlation between entrance air kerma and the Kerma-Area Product was observed, providing a potential method for improved dose estimation and enhanced radiation safety for interventional radiologists.

## 1. Introduction

Interventional radiology (IR) represents the branch of medical radiology that encompasses all diagnostic and therapeutic procedures, both invasive and minimally invasive, performed under the image guidance of radiological techniques [1,2,3,4]. Over the past few decades, IR has significantly advanced, driven by ongoing technological progress that has led to the development of increasingly sophisticated and advanced medical imaging and fluoroscopy equipment [2]. This field is evolving rapidly, complementing traditional surgery due to its undeniable advantages. IR procedures are mainly performed under local anaesthesia, reducing patient discomfort and recovery time [5,6,7]. These minimally invasive techniques, often using percutaneous needle and guidewire access, eliminate surgical incisions, thereby reducing risks. Patients benefit from shorter hospital stays, outpatient treatment options, and lower healthcare costs. Additionally, procedures can be repeated if needed and do not preclude future surgical interventions.

Behind the evolution of IR techniques, cardiologists and interventional radiologists remain among the professional workers to be most exposed to ionizing radiation [8,9,10,11]. Specifically, physicians involved in IR procedures are exposed to a protracted scattered radiation field coming from the patient’s body [12]. The existence of such a chronic exposure scenario may lead to physicians suffering from adverse damage including radiation-induced cataracts and cardiovascular diseases, thus leading to an increased probability and risk incidence of radiation-induced cancer [13,14]. In this context, radiobiological and epidemiological studies have been performed, highlighting both the existence and the underestimation of risk for IR workers, especially in terms of eye lens and brain exposures [15,16,17,18,19,20,21]. Following ICRP recommendations (publications n. 103 in 2007 and n. 118 in 2012) [22,23], the European Council Directive 2013/59/EURATOM [24] has fixed both the whole-body effective dose limit and the equivalent dose limit to the eye lens to 20 mSv/year (lowering the previous limit of 150 mSv/year) for exposed workers such physicians involved in IR practices. The European Council Directive has been transposed in Italy into the 2020/101 Decree Law [25] and its later update 2022/203 Decree Law [26]. The current regulations define dose limits based on ICRP recommendations [22,27], which include tissue weighting factors $w_{t}$ that account for varying radiosensitivity among different tissues, ranging from the most radiosensitive to more radioresistant tissues, such as the brain with a weighting factor of $w_{t}$ = 0.01. Recent concerns about the long-term effects of prolonged ionizing radiation on the operator’s head during interventional procedures have been fueled by studies suggesting a higher mortality rate among involved physicians [13,28,29,30,31]. This evidence suggests the importance of reconsidering the contribution of the brain, traditionally regarded as a highly radioresistant organ, and meninges to the effective dose of exposed workers. As observed, unlike the established limits for the eye lens, today there is currently no established dose limit in the case of the brain and meninges for IR physicians [25,26]. While epidemiological research has not confirmed these findings, understanding the brain’s exposure conditions is essential for improving radiation protection strategies [10,32].

In this work, we found that the absorbed dose to the brain represents a non-negligible component, ranging from 0.81% to 2.18% of the total effective dose per year (20 mSv/year, total effective dose limit) absorbed by physicians involved in angiography for patients’ biliary drainage. Measurements were performed using thermoluminescent dosimeters (TLDs) [33,34] in a controlled IR procedure, simulating the worst exposure scenario of a worker not equipped with radiation protection devices. The methodology involved the use of a plexiglass phantom to simulate the operator’s head, as previously published for the evaluation of the operator equivalent absorbed dose at the eye lens level [35]. Experiments allowed for the evaluation of the absorbed dose distribution in three different regions of the operator’s head (right, left, and anterior) over approximately a month and a half of a normal annual operational IR schedule. Moreover, alongside the existence of a linear relationship between the measured absorbed dose and exposure time, a correlation between dose to the brain and the Kerma-Area Product (KAP) was observed. This finding should be significant as it would allow the estimation of physician absorbed dose to the brain based on a parameter that is consistently known in IR procedures.

## 2. Materials and Methods

TLD-100H chips were used to measure the absorbed dose at the meningeal level for physicians performing biliary draining at the Interventional Radiology Department of Istituto Nazionale Tumori G. Pascale of Naples, Italy. Irradiations were performed simulating interventional procedures on tissue-equivalent phantoms by using the IR clinical C-arm under-couch X-ray machine.

### 2.1. TLD Analysis System

Dosimetric measurements performed in this study were fulfilled using 42 thermoluminescence dosimetry LiF:Mg,Ti chips from Thermo Scientific™ (Waltham, MA, USA) (3.2 × 3.2 × 0.89 mm^3^). The analysis of TLD-100H chips was performed through the Thermo Scientific™ Harshaw TLD™ Model 3500 Manual Reader provided by Harshaw Chemical Company at the Laboratory of Radioactivity (LaRa) of the Department of Physics “E. Pancini” (University of Naples, Federico II). A high-purity (99.995%) nitrogen source was connected to the reader to eliminate chemiluminescence signals that were unrelated to the irradiations. The TLD readout procedure was performed following a previously published procedure: to optimize the thermoluminescence output, the sample was pre-heated for 10 s at 100 °C, followed by a readout up to 260 °C at a heating rate of 5 °C per second [36]. The reading was determined by integrating the signal within the temperature range of 150–250 °C, as the primary peak occurs around 195 °C. Dosimeters were annealed before irradiation using the TLD Annealing Oven “TLD Heat” provided by RadPro (Remscheid, Germany). The 1 h annealing cycle was optimized following a standard procedure from the literature [37]: 15 min heating at 240 °C, 10 min heating at 100 °C, and room temperature cooling. TLDs were characterized to obtain the entrance surface air kerma ($K_{e}$) to the skull by calculating the calibration factor and analyzing each individual sensitivity factor for the specific irradiation energies.

### 2.2. TLD Calibration Factor (CF)

TLDs were calibrated at Istituto Superiore di Sanità (ISS) in Rome (Italy). Dosimeters were calibrated to Cs-137 emitting photons of 662 keV with a dose rate of 0.63 Gy/min. Irradiations were performed through the Gammacell^®^ 40 Exactor irradiator (Best Theratronics, Ottawa, ON, Canada) [38]. TLDs were exposed to known dose values of 0.25 Gy, 0.5 Gy, 1.5 Gy, and 5 Gy, and the electrical charge was collected with a relative error of ±5% for each dosimeter. The calibration curve is reported in the Results section.

### 2.3. Dosimeter Sensitivity Characterization

TLD sensitivity factors ($S_{i}$) were characterized by exposing dosimeters to a uniform dose distribution of 2 Gy. The irradiation was performed at IRCCS Istituto Nazionale Tumori Fondazione G. Pascale of Naples using the clinical linear accelerator Elekta^TM^ Synergy Agility (Stockholm, Sweden) through a pair of opposing fields of 6 MV of photons. TLDs were placed in a water-equivalent plate with a thickness of 0.8 cm. A uniform dose distribution to the targets was ensured by adding below and above the TLD plate a pair of plexiglass slabs (3 cm and 5 cm of thickness, respectively) to consider the dose build-up effect and the backscattered radiation. Each sensitivity factor $S_{i}$ was calculated, assuming a relative error of ±5%, as the ratio of the individual TLD readout $R_{i}$ to the average readout of the entire TLD batch $\bar{R}$:

(1) $$S_{i}=\frac{R_{i}}{\bar{R}}$$

### 2.4. Setup and Irradiation

Experiments conducted in this study were designed to quantify the entrance air kerma to the skull of interventional radiology operators. Specifically, in this study, biliary drainage was selected as the reference procedure, performed using the Siemens Healthcare^TM^ Artis Zeego (Erlangen, Germany) angiography system. This approach allowed for the optimization of the experimental setup to closely replicate the real intervention scenario within a controlled exposure environment. Tissue-equivalent phantoms were used to simulate both the patient and the primary operator: a plexiglass volumetric phantom (13 × 13 × 9 cm^3^) was employed to simulate the head of a right-handed operator, and an anthropomorphic phantom of the abdomen–pelvis region was used to represent the patient [35]. The anthropomorphic phantom’s major axes measured 37, 21, and 42.5 cm.

**Figure 1:**
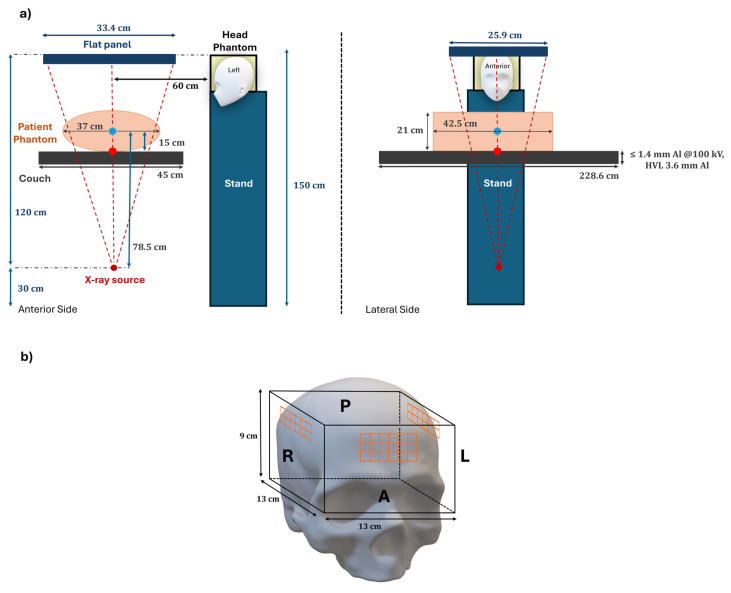
Representation of the experimental setup. (**a**) Anterior and lateral views of the interventional radiology setup reproduced during the experimental phase. (**b**) Reconstruction of the phantom used to simulate the absorbed dose to the operator’s meninges during interventional radiology procedures. The dosimeters measuring the entrance air kerma to the skull (orange) are positioned on the anterior (A), right (R), and left (L) sides of the operator’s head.

The phantom simulating the head of a right-handed primary operator was positioned on a support at a height of 150 cm, representing the median height of an operator bent over during a procedure, and at 60 cm from the central axis of the beam employed in typical IR postero-anterior (PA) X-ray tube angles. The anthropomorphic phantom was placed on the patient couch, between the X-ray source and the flat panel, orthogonally to the radiation beam, to recreate the scattered radiation component from the patient (Figure 1). TLDs were positioned on the plexiglass phantom laterally (12 chips per side) and frontally (18 chips) to separate the entrance air kerma to the skull by the various positions: right (R), left (L), and anterior (A) of the operator’s head. The TLDs were divided into four groups and placed in the same positions for each irradiation in order to investigate the time-dependent variation in entrance air kerma at different cumulative exposure durations (60, 240, 300, and 600 min). The minimum duration of 60 min was selected as it represents a reasonable timeframe to obtain a measurable response from the TLDs while also being consistent with the average KAP values measured for the simplest biliary drainage procedures. The subsequent durations—240, 300, and 600 min—were chosen to represent increasing and realistic fractions of an operator’s typical annual exposure estimated at 4500 min, allowing for the assessment of cumulative dose trends under consistent irradiation conditions. Specifically, a total of 10 irradiations, each lasting 60 min, were performed using the same setup and parameters, ensuring that differences in measured doses were due solely to cumulative exposure time and not to changes in irradiation conditions. This approach corresponds to approximately one and a half months of annual work. During the exposures, the radiological parameters of the angiography system, as reported in the table, were kept constant by the Automatic Exposure Control (AEC) and CARE&CLEARE features (see Table 1), according to the patient’s morphology.

The entrance air kerma to the skull $K_{ei}$ for the i-th TLD-100H was calculated as

(2) $$K_{ei}=\frac{R_{i}}{S_{i}\cdot CF\cdot M(E_{eff})},$$

where $R_{i}$ is the i-th TLD-100H readout subtracted for the background, $S_{i}$ is the sensitivity factor (see Section 2.3), and CF is the calibration factor to Cs-137 (662 keV) (see Section 2.2). $M(E_{eff}$) is the energy correction factor, which takes into account the effective energy of the radiation spectrum to which TLDs have been exposed following the methodology proposed by Nunn et al. [39]. Using the TASMICS spreadsheet [40], it was possible to simulate the radiation beam delivered by the angiographic system, given the fixed radiological exposure parameters (Table 1). Specifically, the effective energy of the beam was found to be 51 keV. Using the tables provided in Davis et al. [36], the average energies of Co-60 (E = 1250 keV), Cs-137 (E = 662 keV), the beam used in the experimental measurements, and various types of beams analyzed in the aforementioned article were considered as a function of the air kerma for a specific energy (K) normalized over the air kerma for Co-60 ($K_{1250keV}$). For the effective energy of the angiographic system considered in the current study, $E_{eff}$ = 51 keV, the normalized air kerma ($K_{51keV}/K_{1250keV}$) was obtained by interpolating the known values by Davis et al. [36], and it was found to be approximately $K_{51keV}/K_{1250keV}\;$= 0.88. Normalizing this value with respect to the air kerma of Cs-137 ($K_{662keV})$ (since the TLDs in the current study were calibrated to this source), the correction factor $M(E_{eff}=51\;keV)$ was determined to be

(3) $$M\left(E_{eff}=51\;keV\right)=\frac{K_{51keV}/K_{1250keV}}{K_{662keV}/K_{1250keV}}=\frac{0.88}{0.97}=0.90$$

## 3. Results and Discussion

The TLDs’ sensitivity factors Si ranged between 0.95 and 1.05, and therefore no dosimeter was rejected following the methodology proposed by Plato and Miklos [41].

**Figure 2:**
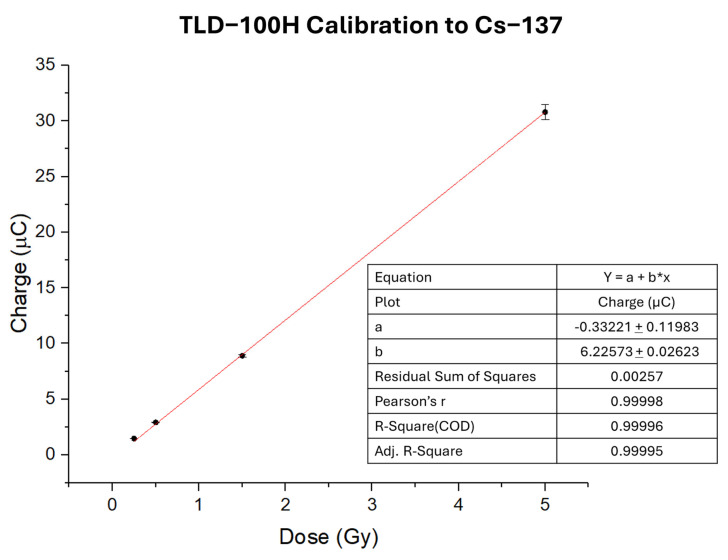
Calibration curve to Cs-137 for TLD-100H dosimeters.

The plot of the collected charge as a function of the irradiation dose from Cs-137 represents the calibration curve (Figure 2). The calibration factor (CF) for TLDs to Cs-137 is evaluated from a linear fit of the calibration curve as the slope of the line as

(4) $${CF}_{Cs-137}=\left(6.22\pm 0.20\right)\frac{\mu C}{Gy}$$

**Figure 3:**
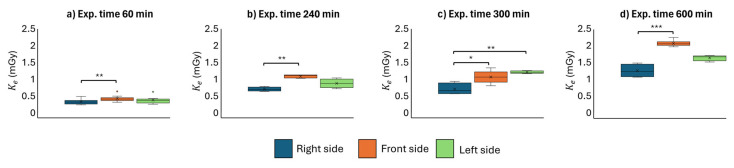
Entrance air kerma to the skull for the right, front, and left sides of the operator’s head, reported as a function of the exposure time: 60 (**a**), 240 (**b**), 300 (**c**), and 600 (**d**) minutes. Statistical differences for each time point are represented in terms of the *p*-value using a Kruskal–Wallis–Dunn test with the following notation: *, *p* < 0.05; **, *p* < 0.01; ***, *p* < 0.001. Non-significant differences are not reported. Relative uncertainties on K_e_ are ±15%.

TLD-100 H dosimeters were used to investigate the entrance air kerma to the skull of the physician during IR procedures for each side of the operator’s head phantom as a function of the exposure time (Figure 3). For each time point, the dose has been evaluated from the average of measures of at least four dosimeters positioned on the three sides of the head phantom.

In Figure 3, the box plots depict the distribution of entrance air kerma to the skull for each side of the operator’s head as a function of exposure time. The frontal and left regions receive significantly higher kerma than the right side of the operator’s head. Specifically, after 60 min of exposure (Figure 3a), the frontal region exhibits a significantly higher kerma than the right side (*p* < 0.01). This difference remains statistically significant (*p* < 0.01) as the exposure time increases up to 240 min. After 300 min of occupational exposure (Figure 3c), the entrance air kerma to the skull on the left side of the operator’s head increases significantly (*p* < 0.01) compared to the right side, although it remains lower than that measured at the forehead (*p* < 0.05). However, differences in exposure between the forehead and left side are not statistically significant, with measured kerma values being comparable within the limits of uncertainty. At 600 min of exposure (Figure 3d), the anterior region receives the highest dose, with a statistically significant difference compared to the right side (*p* < 0.001).

**Figure 4:**
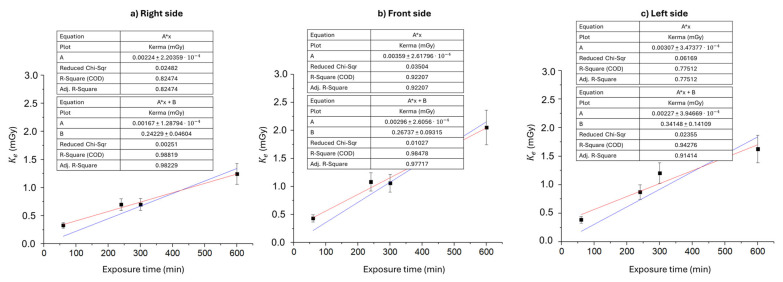
Entrance air kerma to the skull for the operator as a function of the exposure time (60, 240, 300, and 600 min) during the IR procedure. Kerma is evaluated for each side of the head: right (**a**), front (**b**), and left (**c**). Linear fit performed with zero intercept (blue) versus free intercept (red). Relative uncertainties on K_e_ are ±15%.

The graphs presented in Figure 4 demonstrate that, on each side of the operator’s head, the entrance air kerma to the skull is linear with respect to the exposure time. Independently of the chosen linear fit function, the comparison among the three regions of the head highlights that the right side exhibits a slower temporal increase in entrance air kerma (0.0017–0.0022 mGy/min) compared to the other two regions of the head (0.0030–0.0036 mGy/min and 0.0023–0.0032 mGy/min for the front and left sides, respectively). Relative uncertainties on entrance air kerma rates are derived from K_e_ measurements, therefore being ±15%.

This may be due to TLDs on the right side being exposed to lower air kerma values and to a different beam quality with lower mean energy because of the attenuation that happens in the phantom. On the other hand, TLDs on the front and left sides are exposed to similar beams, being closer to the X-ray tube, without any attenuation medium with respect to the patient on the table couch, leading to comparable entrance air kerma rates.

In order to estimate entrance air kerma to the skull per year by IR operators, the measured values were rescaled to the annual estimated operational time of IR procedures. Specifically, considering an average time of 9 min for each IR procedure and a total annual workload of 500 interventional radiology procedures in our institute, the cumulative activity time amounts to 4500 min. As calculated in a previous study by Manna et al. [35], the rescaled entrance air kerma to the skull can be obtained for each region of the operator’s head as follows:

(5) $$K_{60}^{year}=K_{60}\cdot 4500/60$$

where $K_{e}^{60}$ is the mean entrance air kerma to the skull measured for an exposure time of 60 min for each side of the operator’s head (Figure 4a). Using the same approach, the cumulative mean annual entrance air kerma to the skull for each side of the operator’s head should be evaluated, rescaling the measured entrance air kerma to the skull after a 600 min exposure (corresponding to one and a half months of IR procedures):

(6) $$K_{600}^{year}=K_{600}\cdot 4500/600$$

The results for the cumulative mean annual entrance air kerma to the skull for each side of the operator’s head are reported in Table 2. Furthermore, the mean annual entrance air kerma to the skull has been evaluated as $K_{fit}^{year}$ from the linear fitting parameters obtained from the analysis of the mean entrance air kerma to the skull as a function of the exposure time for each side of the operator’s head (Figure 3a–c). To ensure physically consistent modeling, initial linear fits were constrained to pass through the origin, under the assumption that air kerma should be null in the absence of exposure. However, this constraint may introduce a systematic overestimation of the slope, especially due to the influence of the measurement at 60 min, which is characterized by increased uncertainty resulting from the low exposure level. Although background subtraction was applied to each individual TLD measurement, the presence of a residual systematic offset cannot be excluded. Possible sources include instrumental noise, reduced detector sensitivity at low doses, or minor nonlinearities in the TLD dose–response curve at the lower end of the dynamic range. To account for these effects, a more general linear fitting model of the form y=Ax+B was adopted. The resulting intercepts (average B=0.25±0.10 mGy across the three projections) are small, yet they suggest the presence of such residual effects not attributable to the background alone. Notably, the constrained model (y=Ax) systematically yielded higher slope values, leading to a potential overestimation of the measured air kerma by 15–30% in terms of percent difference. For this reason, both fitting models were evaluated—also in subsequent analyses—to provide an estimate of annual occupational exposure that is as representative as possible of the actual experimental conditions. The data in Table 2 show that, for each extrapolation performed, the average annual entrance air kerma to the skull on the left side of a right-handed operator is greater than the annual entrance air kerma to the skull on the right side, in accordance with the scientific literature: studies investigating the occupational hazard of radiation exposure for right-handed interventional radiologists and cardiologists report an increased incidence of brain tumors on the left side of the head [11,30]. The forehead is also highly exposed, with values that are comparable within uncertainties to those measured for the left side. The three different extrapolations carried out highlight that the sum of doses measured by the TLDs following acute exposures is not compatible with the dose absorbed under conditions of prolonged exposure, although there is a linear relationship between the dose absorbed by the brain and the exposure time (Figure 3). The cumulative mean annual entrance air kerma to the skull, evaluated from the mean entrance air kerma measured after 1 hour of exposure $(K_{60}^{year}),$ shows percentage differences of 68.1%, 56.8%, and 65.6% compared to the linear fit extrapolation ($K_{fit}^{year}$) for the right, front, and left sides of the head, respectively. This gap is reduced by comparing $K_{fit}^{year}$ with a cumulative extrapolation obtained from a long exposure of 600 min $\left(K_{600}^{year}\right)$. In fact, the percentage difference between $K_{fit}^{year}$ and $K_{600}^{year}$ for the right, front, and left sides of the head amounts to 17.2%, 11.8%, and 20.8% respectively.

Furthermore, an approximate estimation of the potential contribution of head exposure to the annual effective dose of a radiation worker was performed by assuming that the entrance air kerma measured on various sides of the head corresponds to the absorbed dose to the brain, neglecting attenuation by the skull. Based on the tissue weighting factor for the brain, $w_{t}^{brain}=0.01$, as recommended by ICRP Publication n.103/2007, the partial contribution to the effective dose was estimated using the formula $E=\sum_{i}\left(H_{i}\cdot w_{t}^{i}\right)$. It is acknowledged that the concept of effective dose is intended primarily for scenarios involving uniform or near-uniform whole-body exposure, and its application to partial-body exposures is limited and should be interpreted with caution. In this context, the calculation is intended solely to provide an indicative comparison between localized head exposure and the annual occupational dose limit of 20 mSv. The estimated percentage contributions are reported in Table 3.

The analysis shows that from the cumulative extrapolation of 60 min professional exposures $E(H_{60}^{year})$, the equivalent dose absorbed by the meninges of the IR operator contributes 4.2% of the regulatory limit of 20 mSv for the average annual effective dose of exposed workers (LD 101/2020). A less conservative approach is represented by the cumulative extrapolation of 600 min professional exposures $E(H_{60}^{year})$, which gives 1.9% of the 20 mSv regulatory limit. This last result seems compatible with the linear fitting extrapolation $E(H_{fit}^{year})$ of the brain absorbed dose as a function of the exposure time, which corresponds to 1.6% of the 20 mSv annual effective dose limit for exposed workers. This conservative approach, however, leads to a large overestimation of brain exposure, making it meaningless for radiation protection purposes. A more accurate estimation of brain exposure can be obtained by considering the skull attenuation. With our methodology, it is impossible to perform such an evaluation. However, as reported by Ohno et al. [42], the cranial bone reduced the brain dose to less than half the skin dose: about 48% at the front and less than 9% at the back of the brain. For the three considered entrance kerma values (83.68 mGy, 36.92 mGy, and 30.97 mGy), the absorbed doses to the brain, considering 48% attenuation due to the cranial bone, are 43.51 mGy, 19.20 mGy, and 16.10 mGy, respectively. The corresponding effective doses, applying the tissue weighting factor, are 0.44 mSv, 0.19 mSv, and 0.16 mSv. When compared to the annual regulatory limit of 20 mSv, these values represent 2.18%, 0.96%, and 0.81% of the limit, respectively. These results provide an assessment of brain exposure, highlighting the relatively low but non-negligible contribution of brain irradiation to the overall occupational dose constraint. Although the measured absorbed dose to the brain in our study remains a small fraction of the annual dose limit for occupational exposure (ranging from 0.81% to 2.18% of 20 mSv/year), the potential biological implications warrant consideration, particularly in the context of chronic low-dose exposure over a professional lifetime.

Recent studies have raised concerns about potential associations between prolonged occupational exposure to ionizing radiation and cognitive effects, neuroinflammation, or even increased risk of neurodegenerative diseases, though data remain limited and inconclusive. Notably, the brain is considered relatively radioresistant in terms of deterministic effects, but stochastic effects such as carcinogenesis or subtle functional alterations due to cumulative oxidative stress cannot be entirely excluded.

Our findings, while based on a worst-case scenario, underscore the importance of minimizing even low-level cranial exposure through consistent use of protective measures (e.g., lead caps, ceiling-suspended shields), especially given the increasing procedural volume and longevity of interventional careers. Future research combining dosimetric data with epidemiological or neurofunctional outcomes may help clarify the long-term implications of brain exposure in interventional radiology.

**Figure 5:**
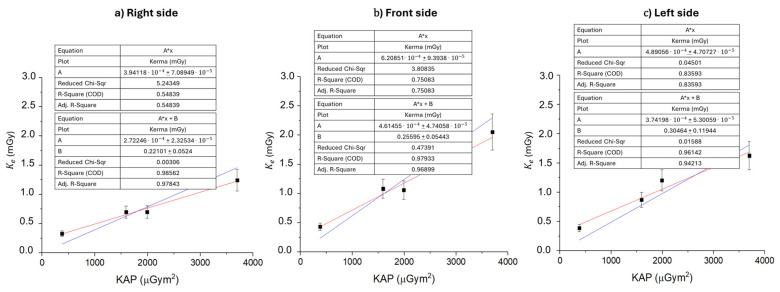
Mean entrance air kerma to the skull (mGy) for the right (a), front (b), and left (c) sides of the operator’s head as a function of the KAP $(\mu Gym^{2}$). KAP is reported for radiological procedures of 60, 240, 300, and 600 min, as the sum of the KAP of continuous hourly exposures. Linear fit performed with zero intercept (blue) versus free intercept (red).

In Figure 5, the relation between the mean entrance air kerma to the skull and the cumulative KAP for prolonged exposures (60, 240, 300, and 600 min) is reported for each side of the operator’s head. The existence of a linear correlation (y = Ax + B, R-Square > 0.95) between dose and KAP, in accordance with the dosimetric outcomes discussed in Figure 3 and Figure 4, opens up potential prognostic scenarios for determining the absorbed dose to the meninges of radiology operators, as the KAP is a parameter available at the end of each interventional procedure. For the same reasons discussed previously, entrance air kerma over KAP values are lower for the right side of the head with respect to the other two sides. The values for the front and left sides are comparable with each other within limits of relative uncertainties of ±15%.

**Figure 6:**
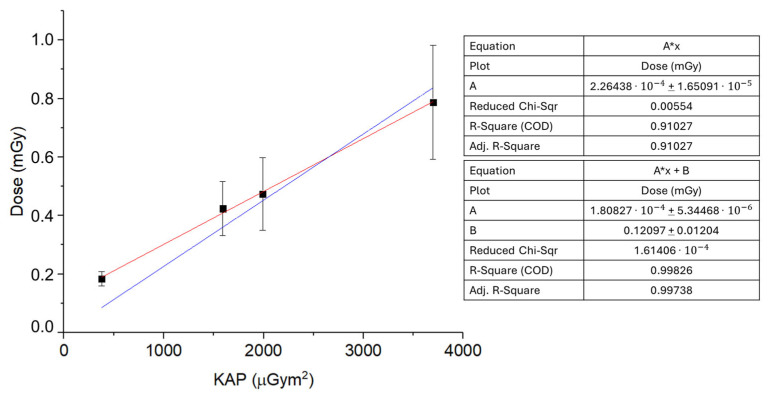
Mean absorbed dose (mGy) to the brain obtained by averaging K_e_ for the right, front, and left sides of the operator’s head as a function of the KAP $(\mu Gym^{2}$), and considering the attenuation of the cranial bone reported by Ohno et al. [42]. KAP is reported for radiological procedures of 60, 240, 300, and 600 min, as the sum of the KAP of continuous hourly exposures. Linear fit performed with zero intercept (blue) versus free intercept (red).

Finally, in Figure 6, an extrapolation of the mean absorbed dose (mGy) to the brain is obtained by averaging K_e_ (see Figure 5) for the right, front, and left sides of the operator’s head as a function of the KAP $(\mu Gym^{2}$), and considering the attenuation of the cranial bone reported by Ohno et al. [42]. The linear fit (y = Ax + B, R-Square > 0.95) returns $A=18.08\pm 0.10\;\mu Gy/Gycm^{2}$, indicating a strong correlation between KAP and the estimated brain dose, demonstrating a strong correlation between KAP and the estimated brain dose.

The elevated dose/KAP value observed in this study, compared to some literature reports [31,43], can be explained by the specific experimental conditions chosen. In our simulation of a biliary drainage procedure, the exposure was deliberately measured in an unshielded environment—without the use of any personal protective equipment such as lead aprons, thyroid collars, or ceiling-suspended shields—to simulate a worst-case scenario. This approach, while providing a conservative upper-bound estimate of operator dose, naturally results in a higher dose per unit KAP. The study was intentionally designed as a worst-case scenario, excluding standard radiation protection measures such as lead caps or ceiling-suspended shields, in order to assess the maximum potential exposure in suboptimal conditions.

Although the fluoroscopic parameters used in biliary drainage typically yield relatively low KAP values per exposure, the cumulative effect of scattered radiation over the course of the procedure—combined with the absence of attenuation from protective devices—leads to an increased dose/KAP ratio. This artificially elevated ratio reflects a scenario in which all potential scatter contributes to operator dose without mitigation.

These findings highlight the critical role of radiation protection in interventional radiology. In routine clinical practice, where dynamic factors (e.g., operator movement, varying beam angles, patient anatomy) and protective measures are present, actual doses are expected to be significantly lower. Therefore, while our results do not directly represent real-world exposures, they underscore the importance of maintaining high safety standards. Future research should focus on quantifying the efficacy of various shielding strategies under realistic procedural conditions to guide the refinement of radiation safety protocols.

## 4. Conclusions

In this study, we determined that the absorbed dose to the brain represents a portion of the total effective dose, accounting for between 0.81% and 2.18% of the annual dose limit (20 mSv/year) received by interventional radiologists performing biliary drainage. Measurements were carried out using thermoluminescent dosimeters during a controlled interventional radiology procedure, simulating the worst exposure scenario of a worker not equipped with radiation protection devices. Experiments allowed for the evaluation of the absorbed dose distribution in three different regions of the operator’s head (right, left, and anterior) over approximately a month and a half of a normal annual operational IR schedule. We found that on each side of the operator’s head, the dose is linear with respect to the exposure time. Specifically, the comparison among the three regions of the head highlights that the forehead exhibits a more rapid temporal increase in absorbed dose with respect to the two lateral regions. The investigation of the distribution of the absorbed dose by the brain measured for each side of the operator’s head also exhibited a dependence on the exposure time, with the anterior side of the head receiving the higher values of absorbed dose. Using both a fitting and a cumulative extrapolation, the average annual dose absorbed by the brain was also defined. Specifically, every case showed how the average annual dose absorbed on the left side of a right-handed operator is greater than the dose absorbed on the right side, in accordance with previous epidemiological studies. Moreover, the three different extrapolations carried out highlight that the sum of doses measured by the TLDs following acute exposures is not compatible with the dose absorbed under conditions of prolonged exposure, although there is a linear relationship between the dose absorbed by the brain and the exposure time. The tissue weighting factor for the brain, $w_{t}^{brain}=0.01$, as recommended by ICRP Publication n.103/2007, makes it possible to consider the contribution of the meninges to the annual effective dose received by exposed workers.

The analysis shows that from the cumulative extrapolation of 60 min professional exposures, the equivalent dose absorbed by the meninges of the IR operator contributes 2.18% of the regulatory limit of 20 mSv for the average annual effective dose of exposed workers (LD 101/2020). A less conservative approach is represented by the cumulative extrapolation of 600 min professional exposures, which gives 0.96% of the 20 mSv regulatory limit. This last result seems compatible with the linear fitting extrapolation of the brain absorbed dose as a function of the exposure time, which corresponds to 0.81% of the 20 mSv annual effective dose limit for exposed workers.

Despite the fact that more studies and measurements are required in order to better understand operators’ head exposure during interventional radiology procedures, this work highlights the importance of evaluating the brain absorbed dose to optimize the radiation protection of exposed workers and comply with the national legislation.

## Figures and Tables

**Table 1:** Radiological parameters automatically set by the Artis Zeego Eco angiography system for the biliary drainage procedure.

Tube Voltage (kV)	Tube Current (mA)	Inherent Filtration (mm Al)	Added Filtration (mm Cu)	Focal Spot (mm^2^)
68	21	2.5	0.6	1.0 × 1.0

**Table 2:** Cumulative mean annual entrance air kerma to the skull $K^{year}$ (mGy) for each side of the operator’s head. The mean annual entrance air kerma to the skull has been evaluated using the mean entrance air kerma to the skull measured after 1 h of exposure ${(K}_{60}^{year})$ and after 1 and a half months of exposure ${(K}_{600}^{year}).$ The same mean annual evaluation ${(K}_{fit}^{year})$ has been carried out using the fitting parameters from Figure 3a–c. Relative expanded uncertainties on TLD-100 H measurements are ∆K/K = 15%.

$K_{60}^{year}$ (mGy)		
Right side 24.16	Front side 31.43	Left side 28.09
$K_{600}^{year}$ **(mGy)**		
Right side 9.32	Front side 15.41	Left side 12.19
$K_{fit}^{year}$ (mGy) [y = A*x + B]		
Right side 7.72	Front side 13.59	Left side 9.66
$K_{fit}^{year}$ (mGy) [y = A*x]		
Right side 10.08	Front side 16.16	Left side 13.82

**Table 3:** Contribution of the equivalent dose absorbed by the meninges to the regulatory limit of 20 mSv for the average annual effective dose of exposed workers (LD 101/2020). The calculation of this percentage contribution has been reported for the cumulative extrapolations of both 60 and 600 min professional exposures ($H_{60}^{year}$, $H_{600}^{year}$) and for the linear fitting extrapolation ($H_{fit}^{year}(y=Ax+B)$) of the brain absorbed dose vs. time of exposure curve in Figure 3.

${E(H}_{60}^{year})$	${E(H}_{600}^{year})$	${E(H}_{fit}^{year})$
**4.2%**	1.9%	1.6%

## Data Availability

All data generated or analyzed during this study are included in this article.

## References

[1] Doherty M. (2019). Value of Interventional Radiology: Past, Present, and Future. Semin. Intervent. Radiol..

[2] Murphy T.P., Soares G.M. (2005). The Evolution of Interventional Radiology. Semin. Intervent. Radiol..

[3] Rösch J., Keller F.S., Kaufman J.A. (2003). The Birth, Early Years, and Future of Interventional Radiology. J. Vasc. Interv. Radiol..

[4] Dotter C.T., Judkins M.P. (1964). Transluminal Treatment of Arteriosclerotic Obstruction: Description of a New Technic and a Preliminary Report of Its Application. Circulation.

[5] Sabharwal T., Fotiadis N., Adam A. (2007). Modern Trends in Interventional Radiology. Br. Med. Bull..

[6] Carlson S.K., Bender C.E., Classic K.L., Zink F.E., Quam J.P., Ward E.M., Oberg A.L. (2001). Benefits and Safety of CT Fluoroscopy in Interventional Radiologic Procedures. Radiology.

[7] O’Brien B., Van Der Putten W. (2008). Quantification of Risk-Benefit in Interventional Radiology. Radiat. Prot. Dosim..

[8] Gerić M., Popić J., Gajski G., Garaj-Vrhovac V. (2019). Cytogenetic Status of Interventional Radiology Unit Workers Occupationally Exposed to Low-Dose Ionising Radiation: A Pilot Study. Mutat. Res./Genet. Toxicol. Environ. Mutagen..

[9] Martin C.J., Magee J.S. (2013). Assessment of Eye and Body Dose for Interventional Radiologists, Cardiologists, and Other Interventional Staff. J. Radiol. Prot..

[10] Zakeri F., Hirobe T., Akbari Noghabi K. (2010). Biological Effects of Low-Dose Ionizing Radiation Exposure on Interventional Cardiologists. Occup. Med..

[11] Reeves R.R., Ang L., Bahadorani J., Naghi J., Dominguez A., Palakodeti V., Tsimikas S., Patel M.P., Mahmud E. (2015). Invasive Cardiologists Are Exposed to Greater Left Sided Cranial Radiation. JACC Cardiovasc. Interv..

[12] Bohari A., Hashim S., Mohd Mustafa S.N. (2020). Scatter Radiation in the Fluoroscopy-Guided Interventional Room. Radiat. Prot. Dosim..

[13] Roguin A., Nolan J. (2021). Radiation Protection in the Cardiac Catheterisation Lab: Best Practice. Heart.

[14] McNamara D.A., Chopra R., Decker J.M., McNamara M.W., VanOosterhout S.M., Berkompas D.C., Dahu M.I., Kenaan M.A., Jawad W.I., Merhi W.M. (2022). Comparison of Radiation Exposure Among Interventional Echocardiographers, Interventional Cardiologists, and Sonographers During Percutaneous Structural Heart Interventions. JAMA Netw. Open.

[15] Braganza M.Z., Kitahara C.M., Berrington De Gonzalez A., Inskip P.D., Johnson K.J., Rajaraman P. (2012). Ionizing Radiation and the Risk of Brain and Central Nervous System Tumors: A Systematic Review. Neuro-Oncol..

[16] Hattori S., Monzen H., Tamura M., Kosaka H., Nakamura Y., Nishimura Y. (2022). Estimating Radiation Exposure of the Brain of a Physician with a Protective Flap in Interventional Radiology: A Phantom Study. J. Appl. Clin. Med. Phys..

[17] Kitahara C.M., Linet M.S., Balter S., Miller D.L., Rajaraman P., Cahoon E.K., Velazquez-Kronen R., Simon S.L., Little M.P., Doody M.M. (2017). Occupational Radiation Exposure and Deaths From Malignant Intracranial Neoplasms of the Brain and CNS in U.S. Radiologic Technologists, 1983–2012. Am. J. Roentgenol..

[18] Picano E., Vano E., Domenici L., Bottai M., Thierry-Chef I. (2012). Cancer and Non-Cancer Brain and Eye Effects of Chronic Low-Dose Ionizing Radiation Exposure. BMC Cancer.

[19] Wenzl T.B. (2005). Increased Brain Cancer Risk in Physicians with High Radiation Exposure. Radiology.

[20] Pugliese M., Amatiello A., Correra M., Stoia V., Cerciello V., Roca V., Loffredo F., Fiore F., La Verde G. (2018). Evaluation of the Current Status of the Eye Lens Radiation Exposure in an Interventional Radiology Department. La Med. del Lav..

[21] Liverani A., Loffredo F., Fiore F., Correra M., La Verde G., Pugliese M. (2018). Evaluation of the Dose Dependence on the Eye Lens from the Position of the Dosimeter for the Operators Exposed in Interventional Radiology. Il Nuovo Cimento C.

[22] ICRP (2012). ICRP Statement on Tissue Reactions/Early and Late Effects of Radiation in Normal Tissues and Organs—Threshold Doses for Tissue Reactions in a Radiation Protection Context. ICRP Publication 118. Ann. ICRP.

[23] International Agency for Research on Cancer (2012). Radiation Volume 100D, a Review of Human Carcinogens. IARC Monographs on the Evaluation of Carcinogenic Risks to Humans.

[24] (2014). Council Directive 2013/59/Euratom of 5 December 2013 Laying down Basic Safety Standards for Protection Against the Dangers Arising from Exposure to Ionising Radiation, and Repealing Directives 89/618/Euratom, 90/641/Euratom, 96/29/Euratom, 97/43/Euratom and 2003/122/Euratom; Official Journal of the European Union. https://eur-lex.europa.eu/eli/dir/2013/59/oj.

[25] (2020). Decreto Legislativo 31 Luglio 2020, n. 101 Attuazione Della Direttiva 2013/59/Euratom, Che Stabilisce Norme Fondamentali Di Sicurezza Relative Alla Protezione Contro i Pericoli Derivanti Dall’Esposizione Alle Radiazioni Ionizzanti, e Che Abroga le Direttive 89/618/Euratom, 90/641/Euratom, 96/29/Euratom, 97/43/Euratom e 2003/122/Euratom e Riordino Della Normativa Di Settore in Attuazione Dell’Articolo 20, Comma 1, Lettera a), Della Legge 4 Ottobre 2019, n. 117; Gazzetta Ufficiale della Repubblica Italiana, Serie Generale n.201 del 12-08-2020—Suppl. Ordinario n. 29. https://www.gazzettaufficiale.it/eli/id/2020/08/12/20G00121/sg.

[26] Disposizioni Integrative e Correttive al Decreto Legislativo 31 Luglio 2020, n. 101, di Attuazione della Direttiva 2013/59/Euratom, che Stabilisce Norme Fondamentali di Sicurezza Relative alla Protezione Contro i Pericoli Derivanti dall’Esposizione alle Radiazioni Ionizzanti, e che Abroga le Direttive 89/618/Euratom, 90/641/Euratom, 96/29/Euratom, 97/43/Euratom e 2003/122/Euratom e Riordino della Normativa di Settore in Attuazione dell’Articolo 20, Comma 1, Lettera a), della Legge 4 Ottobre 2019, n. 117; Gazzetta Ufficiale della Repubblica Italiana, Serie Generale n.2 del 03-01-2023. https://www.gazzettaufficiale.it/eli/id/2023/01/03/22G00207/sg.

[27] International Commission on Radiological Protection (2007). The 2007 Recommendations of the International Commission on Radiological Protection.

[28] Moriña D., Grellier J., Carnicer A., Pernot E., Ryckx N., Cardis E. (2016). InterCardioRisk: A Novel Online Tool for Estimating Doses of Ionising Radiation to Occupationally-Exposed Medical Staff and Their Associated Health Risks. J. Radiol. Prot..

[29] Kunert P., Matyja E., Prokopienko M., Marchel A. (2012). Radiation-Induced Tumours of Meninges. Report on Eight Cases and Review of the Literature. Neurol. Neurochir. Pol..

[30] Roguin A., Goldstein J., Bar O., Goldstein J.A. (2013). Brain and Neck Tumors Among Physicians Performing Interventional Procedures. Am. J. Cardiol..

[31] Ferrari P., Jovanovic Z., Bakhanova E., Becker F., Krstic D., Jansen J., Principi S., Teles P., Clairand I., Knezevic Ž. (2020). Absorbed Dose in the Operator’s Brain in Interventional Radiology Practices: Evaluation through KAP Value Conversion Factors. Phys. Medica.

[32] Roguin A., Goldstein J., Bar O. (2012). Brain Tumours among Interventional Cardiologists: A Cause for Alarm? Report of Four New Cases from Two Cities and a Review of the Literature. EuroIntervention.

[33] D’Avino V., Scarica M., Ametrano G., La Verde G., Manti L., Muto P., Pugliese M., Arrichiello C. (2020). Preliminary Investigation of Performance of Thermoluminescent Dosimeters for Dose Verification in Brachytherapy. Il Nuovo Cimento C.

[34] Liuzzi R., Piccolo C., D’Avino V., Clemente S., Oliviero C., Cella L., Pugliese M. (2020). Dose–Response of TLD-100 in the Dose Range Useful for Hypofractionated Radiotherapy. Dose-Response.

[35] Manna F., De Nardellis G., Carmosino P.A., Ambrosino F., Caruso U., Correra M., Fiore F., La Verde G., Tarotto L., Pugliese M. (2023). Hp(3) vs TLD-100 for Eye Lens Dosimetry in Interventional Radiology Procedures: A Preliminary Study. Eur. Phys. J. Plus.

[36] Davis S.D., Ross C.K., Mobit P.N., Van der Zwan L., Chase W.J., Shortt K.R. (2003). The Response of LiF Thermoluminescence Dosemeters to Photon Beams in the Energy Range from 30 kV X Rays to 60Co Gamma Rays. Radiat. Prot. Dosim..

[37] Guni E., Hellmann I., Wucherer M., Knappe-Kagan P., Hartmann J., Lell M., Adamus R. (2021). Effectiveness of Radiation Protection Caps for Lowering Dose to the Brain and the Eye Lenses. Cardiovasc. Intervent. Radiol..

[38] (2022). Indicazioni Operative per gli Utenti del Servizio di Irraggiamento dell’Istituto Superiore di Sanità con Radiazioni Gamma da Sorgenti di Cs-137; Rapporti ISTISAN 22/11; Istituto Superiore di Sanità, Rome, Italy. https://www.iss.it/documents/20126/6682486/22-15+web.pdf/a4ce8be2-ecef-4843-4a52-21ca99a91728?t=1656923451825.

[39] Nunn A.A., Davis S.D., Micka J.A., DeWerd L.A. (2008). LiF:Mg,Ti TLD Response as a Function of Photon Energy for Moderately Filtered X-ray Spectra in the Range of 20–250 kVp Relative to Co-60. Med. Phys..

[40] Hernandez A.M., Boone J.M. (2014). Tungsten Anode Spectral Model Using Interpolating cubic Splines: Unfiltered X-ray Spectra from 20 kV to 640 kV. Med. Phys..

[41] Plato P., Miklos J. (1985). Production of Element Correction Factors for Thermoluminescent Dosimeters. Health Phys..

[42] Ohno S., Shindo R., Konta S., Yamamoto K., Inaba Y., Chida K. (2024). Radiation Exposure to the Brains of Interventional Radiology Staff: A Phantom Study. Bioengineering.

[43] Hulthén M., Tsapaki V., Karambatsakidou A. (2024). Estimating Brain and Eye Lens Dose for the Cardiologist in Interventional Cardiology—Are the Dose Levels of Concern?. Br. J. Radiol..

